# Halo Gravity Traction Is Associated with Reduced Bone Mineral Density of Patients with Severe Kyphoscoliosis

**DOI:** 10.1155/2016/8056273

**Published:** 2016-11-08

**Authors:** Xiao Han, Weixiang Sun, Yong Qiu, Leilei Xu, Shifu Sha, Benlong Shi, Huang Yan, Zhen Liu, Zezhang Zhu

**Affiliations:** Spine Surgery, Drum Tower Hospital of Nanjing University Medical School, Nanjing, China

## Abstract

*Background*. Halo gravity traction (HGT) is one of the most commonly used perioperative techniques for the treatment of severe kyphoscoliosis. This study was to explore the influence of HGT on the BMD of these patients.* Methods*. Patients with severe kyphoscoliosis treated by preoperative HGT for at least 2 months were included. Patients' BMD were assessed by dual-energy X-ray absorptiometry at lumbar spine (LS, L2–L4) and femur neck (FN) of the nondominant side. The weight and duration of traction, as well as baseline characteristics, were recorded.* Results*. Twenty patients were recruited. The average traction duration was 77.9 ± 13.0 days while the mean traction weight was 39.9% ± 11.1% of total body weight. Remarkable decrease of BMD was observed at LS of 17 (85%) patients and at FN of 18 (90%) patients. After HGT, 75% of patients were found to have osteoporosis, the incidence of which was significantly higher than that before HGT (35%). The correlation analysis revealed BMD reduction was only significantly correlated with the traction duration.* Conclusions*. The current study showed that preoperative HGT can have obvious impact on the BMD. The BMD reduction is associated with traction duration, suggesting that long traction duration may bring more bone mineral loss.

## 1. Introduction

The surgical treatment of severe kyphoscoliosis remains a great challenge with high perioperative complications [1, 2]. To minimize these complications, particularly neurological complication, Stagnara [3] developed the halo gravity traction (HGT) as a preoperative treatment, which was rapidly popularized in Europe. During HGT, the body weight served as a counterforce, with the traction forces being transferred between patient's bed, wheelchair, and walking frame. Preoperative HGT has been proved to be a safe method of applying gradual and sustained traction to maximize surgical correction of kyphoscoliosis. Previous studies demonstrated that 15% to 38% correction in scoliosis and 17% to 35% correction in kyphosis could be achieved by preoperative HGT [4–8]. Sponseller et al. [9] reported that the total complication rate was lower in patients treated with HGT before definitive fusion than those treated without preoperative HGT (27% versus 52%), showing the capability of preoperative HGT in reducing the risk of complications during scoliosis surgery.

Although HGT can effectively lower the incidence of surgical complications, it is noteworthy that HGT-related complications, such as cranial nerve palsy, pin site infection, and pin loosening, have been reported in the literatures [4–8, 10–12]. Besides, patients undergoing HGT are consistently immobilized, thereby making their daily physical motion quite restricted. It is evidenced by previous literatures that bone mineral density (BMD) can be adversely affected due to restriction of physical activities. Houde et al. [13] reported that immobilization of the forearm after hand or wrist surgery can significantly decrease bone mass in the distal radius and ulna, indicating that low bone density may be a consequence of immobilization and muscle weakness. Farr et al. [14] found that low levels of physical activity may compromise bone development. Similarly, bracing has been postulated to reduce the accumulation of bone mass for its restrictive nature which could preclude or discourage physical activity. Cook et al. [15] reported lower BMD values in 11 patients treated with bracing compared with the 30 subjects without bracing. Moreover, generalized low bone mass in both axial and peripheral skeleton has been widely reported in scoliotic patients [15–17]. Therefore, we speculate that the procedure of HGT is likely to have a negative impact on the BMD of patients with severe kyphoscoliosis.

BMD has been reported to be closely associated with the correction outcome of spine deformity. Lee et al. [18] conducted a prospective study on 181 patients and found a positive correlation between BMD and the maximum torque required to insert a pedicle screw, further demonstrating that patients with low BMD have a high risk of pedicle screw loosening. Moreover, low BMD status was associated with high blood loss during scoliosis surgery. Modi et al. [19] performed a prospective study on 44 scoliosis patients, showing that osteoporotic patients could have the risk of high blood loss increased by nine times. Therefore, clarifying the BMD changes during the procedure of HGT is of great importance. However, to the best of our knowledge, the relationship between preoperative HGT and BMD reduction remains obscure. Hence, we performed this prospective study to evaluate the influence of preoperative HGT on BMD and to explore the possible factors predicting the changes of BMD.

## 2. Materials and Methods

This prospective study was approved by the Ethics Committee of the University. From 2012 to 2014, the patients who met the following inclusion criteria were recruited in the study: (1) patients with severe kyphoscoliosis (coronal Cobb angle > 90°); (2) duration of HGT > 2 months; (3) patients undergoing HGT for at least 12 hours per day; (4) traction weight no less than 20% of body weight; (5) patients without physical therapies. Exclusion criteria were (1) patients who had prior spine surgery; (2) any medical conditions that affected bone metabolism; (3) patients with history of recent extra calcium, Vitamin D, caffeine, or hormone intake; (4) patients receiving drug treatment that affected bone metabolism such as bisphosphonate and steroid; (5) patients with neurologic deficit. An informed consent was obtained from the patients or their parents before recruitment. Demographic data included the age, gender, standing height, body weight, diagnosis, duration of traction, and maximum weight of traction. Body mass index (BMI) was calculated by dividing body weight (kg) with standing height squared (m^2^).

### 2.1. Halo Gravity Traction Protocols

Local anesthesia was prescribed to each patient before the application of the halo ring with four to six pins. Pins were tightened to 8–11 Newton-meters (N·m) of torque, depending on the skull size and bone density. The halo ring was suspended a pulley attached to a wheelchair. The initial traction weight was 4 kg. In case of no traction-related symptoms, the traction weight was added by 2 kg per day and finally reached a target weight (about 20% to 60% of the body weight, maximal 15 kg depending on patients' tolerance to HGT). The patients were instructed to undergo HGT more than 12 hours per day, and each patient received at least 1 h sunlight outside during daytime. At night, the weight of the traction was decreased by half. Patients were always in a HGT wheelchair or in HGT in bed, and they were not ambulating until the end of the preoperative HGT. Daily cranial nerve and upper/lower extremity neurological examinations were performed every 8 hours at initial placement and after each increase in traction weight. If complications such as transient nystagmus and upper extremity numbness occurred, the traction weight was reduced to the previous weight. The traction duration was determined by the improvement of the major coronal curve, global kyphosis (GK), pulmonary function, and neurological function. In-traction X-ray films were obtained every 3 weeks until a plateau of correction is reached [10, 20].

### 2.2. Radiographical Measurements

The initial magnitude of major coronal curve and GK were measured on standing posterior-anterior (PA) and lateral radiographs of the whole spine. GK was measured between the maximally tilted upper and lower end vertebrae using the standard Cobb's method [21]. The correction rates for both coronal and sagittal plane were calculated as follows [22]:

(1) Correction  rate=preHGT  angle−postHGT  anglepreHGT  angle×100%.

All measurements were performed using Surgimap software (version 2.0.8; New York, USA).

### 2.3. BMD Measurements

The pre-HGT and post-HGT BMD were measured in each patient using dual-energy radiograph absorptiometry (Lunar-Dpx-IQ, GE, Wauwatosa, WI, USA) at the posteroanterior lumbar spine (vertebrae L2–L4, LS) and the femoral neck (FN) of the nondominant side. In patients with BMD reduction, the reduction rate was calculated as follows:

(2) BMD  reduction  rate  g/cm2·month=Reduction  of  BMD  g/cm2Traction  duration (days)×30.

The *z*-score is defined as the number of standard deviations (SD) from the mean BMD expected for the patient's peers (e.g., for a healthy normal subject matched for age, gender, and ethnic group). Osteoporosis is defined as a BMD loss of 2.5 SD or more below the established mean value at LS or FN [23]. The *z*-scores of the LS BMD and FN BMD were recorded for each patient. Before HGT, patients with and without osteoporosis were categorized as osteoporotic group and nonosteoporotic group, respectively. The BMD measurement protocol for both sites was carried out by the same physician who had been fully trained in the operation of the scanner, the positioning of subjects, and the analysis of results, according to the manufacturer's guidelines.

### 2.4. Statistical Analysis

All data were analyzed using the SPSS version 19.0 (SPSS, Chicago, IL). Patients' demographics were analyzed using the descriptive statistics. Data were presented with mean ± SD. Chi-square test was used to the compare the incidence of osteoporosis before and after HGT. A paired *t*-test was used to compare the differences between pre-HGT BMD and post-HGT BMD. The Student *t*-tests were used to examine intergroup comparisons. The BMD reductions in patients with different etiologies were compared with the one-way analysis of variance (ANOVA). Relationships between BMD reduction and other variables were analyzed using the Pearson correlation analysis. Statistical significance was set at *P* < 0.05.

## 3. Results 

A total of 20 patients (12 males, 8 females) undergoing HGT treatment were recruited in the study (Table 1). The average age was 16.3 ± 7.6 years. The diagnosis included 6 congenital scoliosis (CS), 8 idiopathic scoliosis (IS), and 6 neurofibromatosis type 1 (NF1) cases.

The mean traction weight was 39.9%  ± 11.1% (20.8%–57.1%) of patients' body weight. The average traction duration was 77.9 ± 13.0 days (62–105 days), during which the average coronal curve magnitude decreased from 115.3°  ± 21.4° to 73.9°  ± 17.8° (*P* < 0.001) and the average GK improved from 106.9°  ± 22.3° to 70.9°  ± 13.9° (*P* < 0.001). The mean correction rates for coronal curve and GK were 35.98%  ± 9.72% and 33.27%  ± 6.63%, respectively (Table 1). No HGT-related complications were observed during the application of preoperative HGT.

### 3.1. Changes in BMD

As shown in Table 1, the initial *z*-scores were averaged −2.2 ± 1.4 at LS and −1.5 ± 1.4 at FN. After the completion of HGT, the mean values of *z*-score were significantly reduced to −2.7 ± 1.5 and −1.9 ± 1.5 for LS and FN, respectively. Significant decrease of BMD was observed at LS of 17 (85%) patients and at FN of 18 (90%) patients compared with the initial values (0.654 ± 0.216 g/cm^2^ versus 0.706 ± 0.200 g/cm^2^ for LS; 0.689 ± 0.133 g/cm^2^ versus 0.722 ± 0.125 g/cm^2^ for FN). After HGT, 15 patients were found to have osteoporosis, the incidence of which was higher than that before HGT (75% versus 35%, *P* < 0.05). After excluding the patients without BMD reduction, the mean reduction rates were 0.027 ± 0.018 g/(cm^2^·month) at LS and 0.017 ± 0.009 g/(cm^2^·month) at FN (Table 2).

### 3.2. Correlated Factors of BMD Change

Decrease of LS BMD was observed in 9 (9/12, 75%) male patients and 8 (8/8, 100%) female patients. As shown in Table 2, no significant difference in BMD reduction rate was observed between male and female patients. Taking the etiology of kyphoscoliosis into consideration, we observed a reduction rate of 0.015 ± 0.010 g/(cm^2^·month) in 4 (4/6, 67%) NF1, 0.034 ± 0.024 g/(cm^2^·month) in 5 (5/6, 83%) CS, and 0.029 ± 0.015 g/(cm^2^·month) in 8 (8/8, 100%) IS cases at LS. As shown in Table 3, the association between BMD reduction rates and different etiologies including NF1, CS, and IS was not observed. With respect to the initial BMD status, no significant difference in reduction rate of BMD was observed between osteoporotic and nonosteoporotic group (Table 4). The Pearson correlation analysis revealed that traction duration was positively correlated with BMD reduction in LS and FN (*r* = 0.558, *P* = 0.020 for LS; *r* = 0.581, *P* = 0.012 for FN). However, there was no significant correlation between reduction of BMD and other variables including age, magnitude of the major coronal curve, GK, standing height, patients' weight, BMI, and traction weight (Table 5).

## 4. Discussion 

Halo spinal traction has been widely used for treating severe spinal deformity [24]. Compared with other skeletal tractions, such as halo-femoral, halo-tibial, or halo-pelvic, HGT provides a slow and gradual correction while the patients are awake, making the continuous monitoring of patients' neurological status possible [3, 25]. With the help of preoperative HGT, partial correction of the deformity can be achieved [7, 8]. In the current study, after the completion of HGT, the average correction rates were 35.98% in the coronal plane and 33.27% in the sagittal plane, which were consistent with the results of previous reported study by Sink et al. [5]. Watanabe et al. [4] using HGT for severe scoliosis (≥100°) reported a 28% correction rate after that. Recently, Garabekyan et al. [8] observed a 38% reduction in the main coronal curve and a 25% correction in the sagittal plane with HGT applied to a pediatric population. To be noted, no patient had previous spine surgery or anterior release in this study. Hence, the good correction capability by HGT alone further testified the utility of this technique.

Although the procedure of HGT provides significant benefits for patients with severe kyphoscoliosis, the possible effect of preoperative HGT on patients' BMD should be noticed. Low BMD status was found in many patients with spine deformity [15–17]. Li et al. [26] suggest that the prevalence of low BMD in adolescents with AIS is 20% to 38%. A study by Lammert et al. [17] showed that the BMD were significantly lower in NF1 patients than normal reference population. In the present study, 35% of the patients were also found to be osteoporotic before HGT. Furthermore, lack of weight-bearing physical activity may lead to mechanical unloading (reduced mechanical stress on bone) [27]. According to the theory raised by Wolff [28], bone architecture is largely determined by the relationship between the thickness and number of trabeculae (i.e., the distribution of mass) and the quantitative distribution of mechanical stresses. There is strong evidence that mechanical unloading can suppress the activity of osteoblast and induce the osteoclastic bone resorption, which results in the reduction in bone mass [29, 30]. Lee et al. [31] performed a cross-sectional study of 596 AIS girls and 302 adolescents, showing a positive correlation between inadequate weight-bearing physical activity and low bone mass. On the other hand, Lorentzon et al. [32] reported that increases in physical activity have positive effects on bone mass gains. As the effect of cast immobilization and non-weight-bearing on bone loss has been well established, there used to be a concern that brace treatment for scoliotic patients would adversely affect the accumulation of bone mass [33, 34]. Similarly, we speculated that the procedure of preoperative HGT may have a negative impact on BMD.

To the best of our knowledge, it is the first study to explore the relationship between preoperative HGT and BMD change in patients with severe kyphoscoliosis. The results of the current study showed that, with more than two months of HGT, the BMD decreased in LS and FN with a reduction rate of 0.027 g/(cm^2^·month) and 0.017 g/(cm^2^·month), respectively. Since the patients' physical activity was restricted by the wheelchair traction, inadequate weight-bearing physical activity was inferred to be the main reason for the reduction in BMD [35]. We further analyzed the factors associated with BMD reduction including the traction duration, traction weight, and the demographic parameters, identifying traction duration as the key factor related to BMD reduction. For patient undergoing HGT, sitting on the wheelchair makes it difficult to take exercise. Therefore, our results showed that the reduction of BMD was positively correlated with traction duration, further confirming the fact that the bone loss was caused by the inadequate physical activity.

Low BMD implied high risks of osteoporosis and fracture, which could make the fixation difficult and result in increased risks of correction loss and implant failure [36, 37]. The pedicle screw pullout strength was showed to be highly correlated with BMD in the reported literatures [18, 37, 38]. Okuyama et al. [38] showed a significant difference of the mean BMD between the patients with and without screw loosening. Lee et al. [18] also found that the mean magnitude of torque in the osteoporotic patients was significantly lower than that in nonosteoporotic patients. Besides, patients with lower BMD were reported to have higher intraoperative blood loss according to Modi et al.'s study [19]. Therefore, improving bone quality before surgery is one of the key factors to achieve a good surgical outcome. In addition to physical activity, many studies have shown that calcium supplementation, vitamin D intake, and sunshine exposure could improve BMD [39, 40]. Thus, to minimize the bone loss in patients undergoing preoperative HGT, it is recommended that patients should get more calcium supplementation, vitamin D intake, and outside sunlight exposure. More physical activity was also recommended for these patients. It is noteworthy that scoliotic patients were instructed to walk by means of an overhead walking frame at some other institutions [6–10]. They were able to walk on a treadmill while in the frame and no osteopenia cases were reported with walking HGT frames [41]. Since halo walker device makes it more convenient to participate in exercises compared with wheelchair device, it may be a better option to minimize the impact of HGT on BMD of patients. However, this assumption needs to be validated in the future studies.

One limitation of this study is that each patient had only one follow-up BMD data, which made it difficult to assess the precise trend of BMD reduction along with traction duration. Nevertheless, this study was the first to analyze the change of BMD in patients with severe kyphoscoliosis undergoing HGT. We found that the procedure of preoperative HGT did reduce the BMD in patients with severe kyphoscoliosis and this reduction was significantly correlated with traction duration. The results suggested that spine surgeon should be aware of this defect and the traction duration should be determined by the compromise between the improvement in spinal deformity and the loss of bone mineral.

## 5. Conclusions

The procedure of HGT has a negative impact on the BMD of patients with severe kyphoscoliosis. Moreover, the BMD reduction is associated with traction duration, suggesting that long traction duration could bring more bone mineral loss.

## Figures and Tables

**Table 1:** Demographics of the cohort (*n* = 20).

	Pre-HGT	Post-HGT	*P*
Age (years)	16.3 ± 7.6	N/A	—
Traction duration (days)	77.9 ± 13.0	N/A	—
Traction weight (%)	39.9 ± 11.1	N/A	—
Height (m)	1.37 ± 0.21	1.38 ± 0.21	0.794
Weight (kg)	33.7 ± 13.3	34.1 ± 13.4	0.612
BMI (kg/m^2^)	16.97 ± 3.04	17.18 ± 3.00	0.235
Major coronal Cobb angle (°)	115.3 ± 21.4	73.9 ± 17.8	<0.001
GK (°)	106.9 ± 22.3	70.9 ± 13.9	<0.001
*z*-score of LS BMD	−2.2 ± 1.4	−2.7 ± 1.5	<0.001
*z*-score of FN BMD	−1.5 ± 1.4	−1.9 ± 1.5	<0.001
LS BMD (g/cm^2^)	0.706 ± 0.200	0.654 ± 0.216	<0.001
FN BMD (g/cm^2^)	0.722 ± 0.125	0.689 ± 0.133	<0.001

“HGT” denotes the halo gravity traction.

N/A: not applicable.

“BMI” denotes body mass index.

“GK” denotes global kyphosis.

“LS” denotes lumbar spine (L2–L4).

“FN” denotes femoral neck of the nondominant side.

**Table 2:** Reduction rates of BMD between males and females.

		All	Male	Female	*P*
LS	*n*	17	9	8	
	BMD (g/[cm^2^·month])	0.027 ± 0.018	0.023 ± 0.010	0.032 ± 0.023	0.286
FN	*n*	18	11	7	
	BMD (g/[cm^2^·month])	0.017 ± 0.009	0.019 ± 0.009	0.015 ± 0.007	0.374

“LS” denotes lumbar spine (L2–L4).

“FN” denotes femoral neck of the nondominant side.

“*n*”: number of patients with BMD reduction.

*P* < 0.05: significant difference between males and females.

**Table 3:** Reduction rates of BMD among CS, NF1, and IS.

		NF1	CS	IS	*P*
LS	*n*	4	5	8	
	Reduction rate (g/[cm^2^·month])	0.015 ± 0.010	0.034 ± 0.024	0.029 ± 0.015	0.274
FN	*n*	6	4	8	
	Reduction rate (g/[cm^2^·month])	0.020 ± 0.007	0.015 ± 0.005	0.016 ± 0.011	0.610

“LS” denotes lumbar spine (L2–L4).

“FN” denotes femoral neck of the nondominant side.

“NF1” denotes neurofibromatosis type 1.

“CS” denotes congenital spinal deformity.

“IS” denotes idiopathic spinal deformity.

“*n*”: number of patients with BMD reduction.

**Table 4:** Reduction rates of BMD between nonosteoporotic and osteoporotic groups.

		Nonosteoporotic group (*z*-score > −2.5SD)	Osteoporotic group (*z*-score ≤ −2.5SD)	*P*
LS	*n*	10	7	
	BMD (g/[cm^2^·month])	0.025 ± 0.016	0.030 ± 0.020	0.600
FN	*n*	11	7	
	BMD (g/[cm^2^·month])	0.015 ± 0.008	0.020 ± 0.009	0.272

“LS” denotes lumbar spine (L2–L4).

“FN” denotes femoral neck of the nondominant side.

“*n*”: number of patients with BMD reduction.

*P* < 0.05: significant difference between males and females.

**Table 5:** Correlations analysis of BMD reduction.

	Age	Coronal Cobb angle	GK	Standing Height	Weight	BMI	Traction duration	Traction weight
LS BMD (*n* = 17)	−0.168	0.191	−0.155	−0.185	0.234	−0.149	0.558^*∗*^	0.133
FN BMD (*n* = 18)	0.147	0.058	−0.471	0.213	0.112	−0.027	0.581^*∗*^	0.124

“LS” denotes lumbar spine (L2−L4).

“FN” denotes femoral neck of the nondominant side.

“GK” denotes global kyphosis.

“*n*”: number of patients with BMD reduction.

“*∗*” *P* < 0.05.
